# Ketamine Reduces Avoidance Responses During Re-Exposition to Aversive Stimulus: Comparison Between (*S*)-Isomer and Racemic Mixture

**DOI:** 10.3390/brainsci14121291

**Published:** 2024-12-22

**Authors:** Clarissa A. Moura, Anne N. de Sousa-Silva, Ana Lívia Mesquita Soares, Carina I. de Oliveira Torres, Hindiael Belchior, Edilson D. da Silva Jr, Elaine C. Gavioli

**Affiliations:** 1 Department of Biophysics and Pharmacology, Federal University of Rio Grande do Norte, Natal 59078-900, Brazil; clari.almeidamoura22@gmail.com (C.A.M.); anne.nathalia@gmail.com (A.N.d.S.-S.); ana.livia.soares.616@ufrn.edu.br (A.L.M.S.); carinaiona.torres@gmail.com (C.I.d.O.T.); edsjunior9@gmail.com (E.D.d.S.J.); 2 Department of Physical Education, Federal University of Rio Grande do Norte, Av. Senador Salgado Filho, Campus Universitário—Lagoa Nova, Natal 59078-900, Brazil; hindiael@gmail.com

**Keywords:** ketamine, aversive memory, animal model, mouse, depression, anxiety

## Abstract

Background/Objectives: Recent studies have investigated the effects of ketamine on fear memory in animals. However, it is unclear if ketamine might affect avoidance memory and emotional behaviors concomitantly. In this study, we compared the effects of (*R*,*S*)- and (*S*)-ketamine in modulating avoidance responses, depression- and anxiety-related behaviors in stressed mice. Methods: Mice were previously exposed to inescapable footshock stress, and 24 h later, they were trained in the active avoidance task. (*R*,*S*)-ketamine or (*S*)-isomer was administered 1 h prior to re-exposition to the active avoidance task. Three hours after drug administration, mice were tested in the tail suspension, followed by the open field test. Results: Neither form of ketamine affected avoidance memory retrieval, while (*S*)-ketamine, and tangentially, (*R*,*S*) reduced avoidance responses during re-exposition to aversive stimulus. In the tail suspension test, (*R*,*S*)- and (*S*)-ketamine equally evoked antidepressant effects. In the open field test, the racemic mixture, but not (*S*)-ketamine, induced anxiolytic actions. Conclusions: These findings reinforce the therapeutic potential of ketamine for the treatment of stress-related disorders, with (*R*,*S*)-ketamine being more effective in simultaneously inducing antidepressant and anxiolytic responses and reducing avoidance responses in stressed mice.

## 1. Introduction

Originally developed as an anesthetic, ketamine has recently emerged as an innovative approach for treating treatment-resistant depression. However, its effects on aversive memories remain a topic of debate [1]. Conditioned fear memory, an adaptive response observed across various animal species, plays a crucial role in survival; however, in humans, an excessive expression of such memories is linked to mental disorders, including depression, anxiety, and post-traumatic stress disorder (PTSD) [2,3]. Thus, the exacerbation of aversive memories is a challenge in treating stress-related disorders, highlighting the potential for medications to modulate these memories effectively.

Stress, a central factor in many mental disorders, is known to enhance the learning of conditioned fear, thereby reinforcing the consolidation of aversive memories [4,5]. In conditions like PTSD, fear memory extinction is often impaired, leading to difficulties in distinguishing between threatening and non-threatening stimuli. This impairment may manifest as an exaggerated stress response to a conditioned stimulus, even in the absence of the original aversive stimulus [6,7]. In major depressive disorder, the acquisition of aversive memories tends to be facilitated over positive ones, a process likely influenced by increased amygdala responsiveness [8]. Evidence indicates that reduced input from the medial prefrontal cortex to the amygdala may contribute to the facilitation of aversive memories and reduction of fear extinction, a pattern commonly observed in PTSD [9].

The ability of (*R*,*S*)-ketamine and its (*S*)-enantiomer to rapidly alleviate depressive symptoms in humans and to reverse depressive-like behaviors in animal models is widely documented. In a comprehensive review, Wei and colleagues [10] provided a historical overview of antidepressant evidence for ketamine and its enantiomers in both rodent and human studies. In a learned helplessness model in rats, Koike et al. [11] reported that (*R*,*S*)-ketamine (10 mg/kg) produced rapid antidepressant-like effects that lasted for at least 72 h after a single dose, marking the first evidence of sustained antidepressant action in a preclinical setting. Similarly, in a chronic unpredictable mild stress model, a single dose of (*R*,*S*)-ketamine (10 mg/kg) induced rapid and enduring antidepressant effects in rodents [12,13,14]. The effects of (*S*)-ketamine in animal models of depression have also been extensively documented, demonstrating efficacy and long-lasting actions at dose ranges comparable to the racemic mixture [15,16,17].

The primary effect ketamine on the brain is its antagonism of the NMDA receptor, which leads to increased glutamate levels in the synaptic cleft [18]. This rise in glutamate is associated with enhanced synaptic plasticity, largely through the activation of ionotropic receptors such as AMPA, which may contribute to improved cognition and emotional regulation in key areas such as the hippocampus, amygdala, and prefrontal cortex [19]. Based on this, we hypothesized that (*R*,*S*)-ketamine treatment and/or its (*S*)-isomer could modify avoidance responses, which are often exaggerated and persistent in depression and PTSD.

In this study, aiming to investigate the effects of ketamine (*R*,*S*)- and (S)-isomer in avoidance responses, anxiety, depression, and locomotion, mice were first subjected to inescapable stress, and later, they were tested in the active avoidance task, followed by tail suspension and open field tests. This experimental timeline was designed to investigate whether (*R*,*S*)- and (*S*)-ketamine influence avoidance responses and emotional behaviors simultaneously or not. Additionally, we compared the effects of (*R*,*S*)-ketamine and its (*S*)-enantiomer (10 mg/kg for each), administered 1 h before re-exposition to a traumatic event, to simulate a therapeutic intervention akin to that in depressive and PTSD patients.

## 2. Materials and Methods

### 2.1. Animals

Male Swiss mice (12 weeks old, 35–40 g) obtained from the animal facility of the Federal University of Rio Grande do Norte were used in this study. The animals were housed in cages measuring 41 × 34 × 16 cm, a maximum number of eight mice per cage, under controlled environmental conditions (temperature: 23 ± 1 °C; light cycle: 12:12 h light–dark cycle, lights on at 6:00 a.m.). Food and water were provided ad libitum, except during the behavioral testing sessions. All behavioral tests were performed between 08:00 and 16:00 h. The experimental procedures were performed in accordance with the recommendations of Brazilian Law No. 11.794/2008 after approval by the Ethics Committee on the Use of Animals of the Federal University of Rio Grande do Norte (License No. 334.016/2023; approval date 11 May 2023). Considering sex differences in the neuroendocrine response to acute stressors [20], only male mice were employed. In this study, 44 mice were distributed in 4 groups: control and (*R*,*S*)-ketamine (13 animals/per group), and control and (*S*)-ketamine (9 animals/per group). All animals were exposed to inescapable stress. From them, 21% of mice were excluded after active avoidance training due to easily learning to escape (details see: Section 2.3.2). Then, in the (*R*,*S*)-ketamine series, 09 drug-treated and 11 control mice were used, while in the (*S*)-ketamine series, 07 animals/per group were employed.

### 2.2. Drugs and Treatments

(*R*,*S*)-ketamine 10 mg/kg (concentration 50 mg/ml, Syntec do Brasil Ltd.a, Barueri, Brazil) and (*S*)-ketamine 10 mg/kg (concentration 50 mg/ml, Cristália, Itapira, Brazil) were diluted in 0.9% NaCl and acutely administered intraperitoneally (i.p.) in mice. The control group received an equivalent volume (10 ml/kg) of the same vehicle. The doses of (*R*,*S*)-ketamine and (*S*)-ketamine were selected based on previous studies demonstrating antidepressant effects in behavioral despair tests [11,17].

### 2.3. Behavioral Tests

#### 2.3.1. Inescapable Stress by Footshock

**Figure 1:**
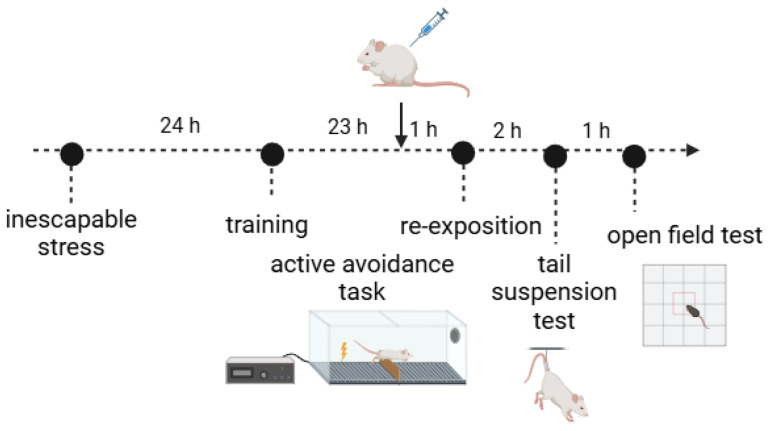
Schematic representation of the experimental design. Mice were firstly exposed to inescapable footshock stress. Twenty-four hours later, they were subjected to the active avoidance task, which consisted of training and re-exposition sessions. One hour before the reexposition session of the active avoidance task, animals were treated with (*R*,*S*)-ketamine, (*S*)-ketamine (both at a dose of 10 mg/kg), or vehicle (ip). Approximately 3 h after treatment, the animals underwent the tail suspension test, followed by the open field test. Figure created in BioRender.com.

Mice were exposed to an inescapable electric footshock session 24 h before behavioral evaluation. Inescapable stress was performed as previously described by Holanda et al. [21]. Mice were placed in an acrylic shuttle-box (50 × 30 × 30 cm) with a stainless-steel grid floor (0.3 × 1 cm), attached to an electric shock generator (AVS Projetos, Ribeirão Preto, Brazil). The box was divided into two compartments by a guillotine door (12 × 25 cm). The inescapable stress session was conducted with the animal placed on one side of the shuttle-box (door closed). Each mouse received 180 randomly administered footshocks (0.5 mA; each cycle: 1–10 s duration, 10 s intervals), and during the inescapable session, a light (30 lux) inside the box remained on (Figure 1).

#### 2.3.2. Active Avoidance Test

The active avoidance test was performed on two consecutive days, as previously described by Gomez et al. [22], following adaptations, 24 h after exposure to inescapable stress (Figure 1). In this test, mice were individually placed in the shuttle-box on two consecutive days for 30 escapable electric footshocks (0.5 mA, shock duration 1–10 s, mean interval of 20 s between shocks), and each shock was preceded by a light signal (30 lux, light-on 1 s before shock, light duration 3 s); in this case, the door located in the center of the shuttle-box remained open. During the first active avoidance session (training), animals that successfully escaped from the shock by crossing to the opposite side of the box through the door more than 10 times were excluded from the study (21% of the animals). The remaining animals were randomly assigned to drugs or control groups. On day 2 (re-exposition session), 60 min after treatment administration, mice were again exposed to the light signal before 30 footshocks (0.5 mA, shock duration 1–10 s, mean interval of 20 s between shocks), and the following parameters were assessed on both days: latency (s) to escape and number of escapes (i.e., the frequency of crossing to the opposite side of the box). The mobility of the animals between the sides of the box was recorded manually by an experienced observer who was blinded to the treatment conditions.

#### 2.3.3. Tail Suspension Test

The tail suspension test was conducted as described by Steru et al. [23], with modifications. This test was performed approximately 3 h after drug administration, on the second day of the active avoidance test (Figure 1). During the test, mice were suspended by the tail using adhesive tape affixed 1 cm from the tip of the tail, with the animal positioned at a height of more than 50 cm above the ground. Each mouse remained suspended for 6 min, and a trained observer manually recorded the immobility time (s) (i.e., did not struggle) during the last 4 min using a stopwatch. The tail suspension test was performed individually and only once.

#### 2.3.4. Open Field Test

The open field test was performed in an arena (40 × 40 × 40 cm) approximately 4 h after treatment with (*R*,*S*)-ketamine, (*S*)-ketamine, or saline (Figure 1). The test was performed in a soundproof room under dark conditions, and the arena was cleaned with a 5% ethanol solution between animals. Each mouse was placed in the center of the open field, and the exploration behavior over 30 min was recorded by an infrared video camera connected to a computer and subsequently analyzed using Anymaze^®^ video tracking software (Stoelting Co., Wood Dale, IL, USA). This test aimed to assess spontaneous locomotion and anxiety-related behaviors by the exploration to the central area. The following parameters were recorded: total distance moved (in meters) over 30 min, and time spent (in seconds) and distance moved in the center (20 × 20 cm) zone of the arena during the first 10 min of exposition. The % center time was calculated by dividing the time spent in the center by 600. The % center distance was obtained by dividing the distance moved in the center zone by the total distance moved in 10 min. Increased exploration time in the central zone indicates anxiolytic-like behaviors, while a decrease suggests anxiogenic-related behavior [24].

### 2.4. Statistical Analysis

Data are presented as the mean ± standard error of the mean (SEM). Normality and homogeneity of variance were assessed with the Kolmogorov–Smirnov test prior to applying parametric statistical tests. Outliers were identified using the ROUT method (Q = 1%) and excluded from the analyses when detected. An unpaired *t*-test was used to compare differences between drug treatment and its respective control group. A repeated measures (RM) ANOVA (mixed effects), followed by the Bonferroni or Tukey’s post hoc test when appropriate, was employed when comparing more than one independent factor, such as sessions and treatment. The Pearson’s correlation coefficient was used to evaluate the linear relationships between behavioral variables. Differences were considered statistically significant at *p* < 0.05. Statistical analysis was conducted using GraphPad Prism Software version 9.0 for Windows (GraphPad Software Inc., San Diego, CA, USA).

## 3. Results

### 3.1. Effects of (R,S)-Ketamine and (S)-Ketamine on the Active Avoidance Task in Stressed Mice

**Figure 2:**
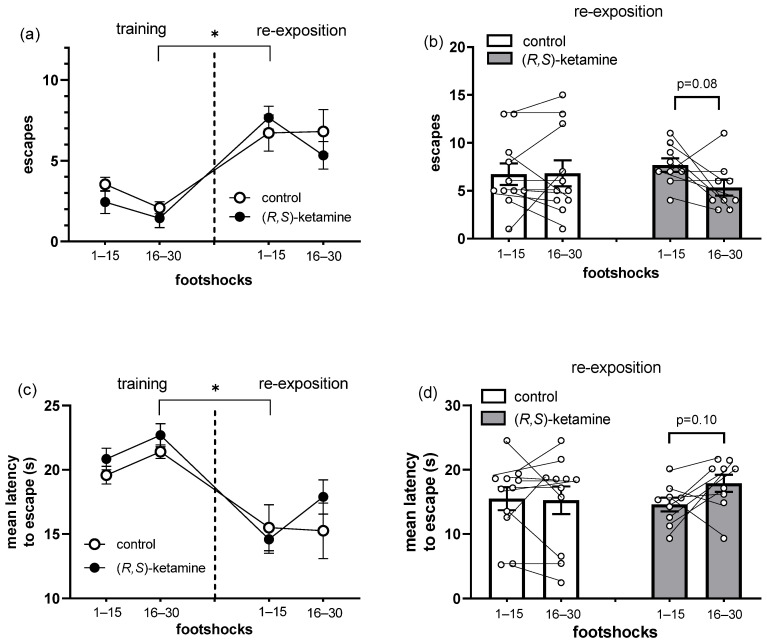
Effects of (*R*,*S*)-ketamine, 10 mg/kg, ip, administered 1 h before the re-exposition session, on the active avoidance task performed in stressed mice. (**a**) Frequency of and (**c**) mean latency to escape from the electrified side of the box in the training and re-exposition sessions, and (**b**) frequency of and (**d**) mean latency to escape in the re-exposition session of the active avoidance task. Data represent the mean ± SEM, while dots represent individual performance. (*R*,*S*)-ketamine: *n* = 9, and control: *n* = 11. (**a**,**c**) RM ANOVA, Tukey’s test: * *p* < 0.05 vs. 16–30 training session; (**b**,**d**) paired *t*-test: footshocks 16–30 vs. 1–15.

**Figure 3:**
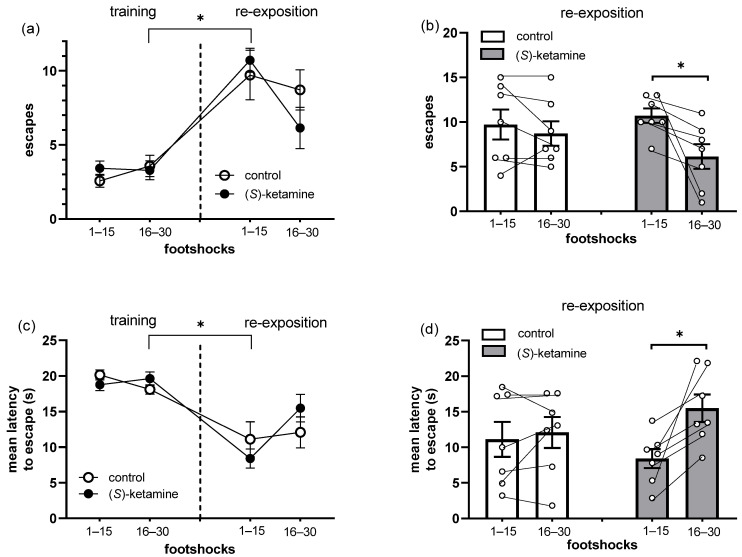
Effects of (*S*)-ketamine, 10 mg/kg, ip, administered 1 h before the re-exposition session, on the active avoidance task performed in stressed mice. (**a**) Frequency of and (**c**) mean latency to escape from the electrified side of the box in the training and re-exposition sessions, and (**b**) frequency of and (**d**) mean latency to escape in the re-exposition session of the active avoidance task. Data represent the mean ± SEM, while dots represent individual performance. (*S*)-ketamine: *n* = 7, and control: *n* = 7. (**a**,**c**) RM ANOVA, Tukey’s test: * *p* < 0.05 vs. 16–30 training session; (**b**,**d**) paired *t*-test: footshocks 16–30 vs. 1–15.

Twenty four hours after the exposure to inescapable stress, mice were tested to evaluate the impact of treatment with (*R*,*S*)-ketamine (Figure 2) and (*S*)-ketamine (Figure 3) on the retrieval of conditioned avoidance responses. When comparing performance between training and re-exposition sessions in the active avoidance task, the control group exhibited a significant increase in the escape frequency and a reduction in the mean escape latency (Figure 2a,c and Figure 3a,c), thus suggesting memory retrieval. The treatment with (*R*,*S*)-ketamine 10 mg/kg did not affect aversive memory recall, as observed when comparing the performance in the number of escapes (Figure 2a, RM ANOVA, sessions factor: F(3, 75) = 17.6, *p* < 0.0001) and the latency to escape (Figure 2c, RM ANOVA, sessions factor: F(3, 75) = 11.9, *p* < 0.0001). To better understand the dynamics of animal behavior during the re-exposition session, this moment was split into two parts based on the number of footshocks: 1–15 and 16–30 footshocks. Importantly, in the second part of the re-exposition session, the control group behaved similarly to the first part. Interestingly enough, the administration of (*R*,*S*)-ketamine 1 h before the re-exposition session trended to reduce the number of escapes (Figure 2b, paired *t*-test, t(8) = 2.0, *p* = 0.08) and to increase the latency to escape from the footshock (Figure 2d, paired *t*-test, t(8) = 1.8, *p* = 0.10) in the second part of the session compared with the first, thus reducing avoidance responses in the re-exposition to the aversive stimulus.

Figure 3 depicts the effects of (*S*)-ketamine in the retrieval of an associative aversive memory in mice. We observed that (*S*)-ketamine did not affect memory retrieval, since a significant increase in the number of escapes (Figure 3a, RM ANOVA, sessions factor: F(3, 51) = 21.7, *p* < 0.0001) and a reduction in the latency to escape (Figure 3c, RM ANOVA, sessions factor: RM ANOVA, sessions factor: F(3, 51) = 17.2, *p* < 0.0001) were detected between training and re-exposition sessions. However, (*S*)-ketamine in the re-exposition session significantly reduced the frequency of escapes (Figure 3b, paired *t*-test, t(6) = 3.2, *p* = 0.02) and increased the latency to escape from the electrified side of the box (Figure 3d, paired *t*-test, t(6) = 3.7, *p* = 0.01) in the second part of the re-exposition session compared to the first. Therefore, acute treatment with (*S*)-ketamine significantly reduced avoidance responses during re-exposition to aversive stimulus.

### 3.2. Effects of (R,S)-Ketamine and (S)-Ketamine on the Tail Suspension Test in Mice After Inescapable Stress

**Figure 4:**
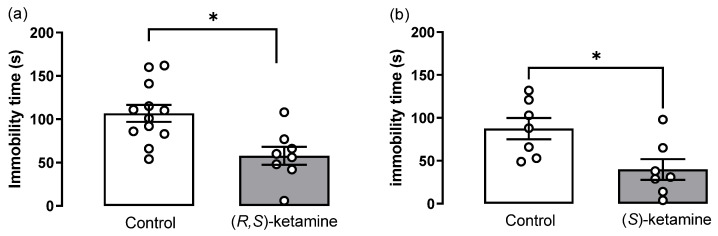
Effects of (*R*,*S*)-ketamine and (*S*)-ketamine (both 10 mg/kg, ip, 3 h before testing) on the immobility time of stressed mice assessed in the tail suspension test. Immobility time (s) in (**a**) (*R*,*S*)-ketamine-treated (*n* = 8) and control (*n* = 11) mice, and (**b**) (*S*)-ketamine-treated (*n* = 7) and control (*n* = 7) mice. Bars represent the mean ± SEM, whereas dots represent individual performance. Unpaired *t*-test: * *p* < 0.05 vs. control.

We next evaluated the effects of (*R*,*S*)- and (*S*)-ketamine in the immobility behavior of stressed mice (Figure 4). The treatment with (*R*,*S*)-ketamine 10 mg/kg significantly reduced the immobility time compared to the control group (Figure 4a, *t*-test: t(17) = 3.1, *p* = 0.006). Similarly, the treatment with (*S*)-ketamine 10 mg/kg significantly reduced immobility time compared to the respective controls (Figure 4b, *t*-test: t(12) = 2.7, *p* = 0.018).

### 3.3. Effects of (R,S)-Ketamine and (S)-Ketamine on the Open Field Test in Mice After Inescapable Stress

**Figure 5:**
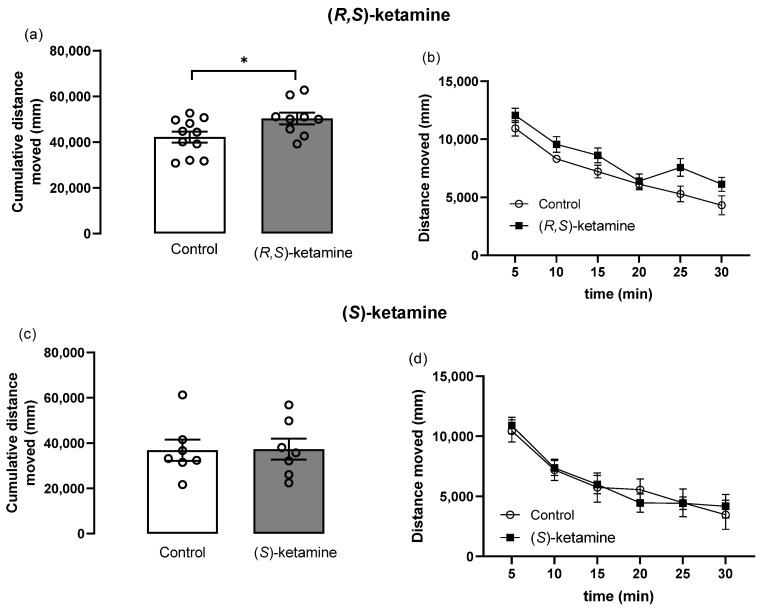
Effects of (*R*,*S*)-ketamine and (*S*)-ketamine (both 10 mg/kg, ip, 4 h before testing) on the spontaneous locomotion of mice previously exposed to inescapable stress. Total distance moved and distance moved in 5 min blocks over time in the (*R*,*S*)-ketamine (**a**,**b**) and (*S*)-ketamine (**c**,**d**) groups. Bars represent the mean ± SEM, while dots represent individual performance. (*R*,*S*)-ketamine: *n* = 9, control: *n* = 11; (*S*)-ketamine: *n* = 7, control: *n* = 7. * *p* < 0.05 vs. control, unpaired *t*-test.

Figure 5 illustrates the distance moved by stressed mice treated with racemic mixture and (*S*)-ketamine in the open field test. (*R*,*S*)-ketamine, but not (*S*)-ketamine, significantly increased the total distance moved compared to control groups ((*R*,*S*)-ketamine: Figure 5a, *t*-test: t(18) = 2.3, *p* = 0.03; Figure 5b, two-way ANOVA: time factor: F(5, 108) = 26.4, *p* < 0.0001, treatment factor: F(1, 108) = 14.1, *p* = 0.0003, Bonferroni post-hoc: *p* > 0.05; (*S*)-ketamine: Figure 5c, *t*-test: *p* > 0.05; Figure 5d, two-way ANOVA: time factor: F(5, 72) = 15.2, *p* < 0.0001, Bonferroni post-hoc: *p* > 0.05). Considering that hyperlocomotion may bias the interpretation of the immobility in the tail suspension test, a correlation was calculated in (*R*,*S*)-ketamine-treated mice. The Pearson’s test did not show any significant correlation between the immobility time in the tail suspension test and the total distance moved in the open field (r^2^ = 0.20, *p* = 0.14).

**Figure 6:**
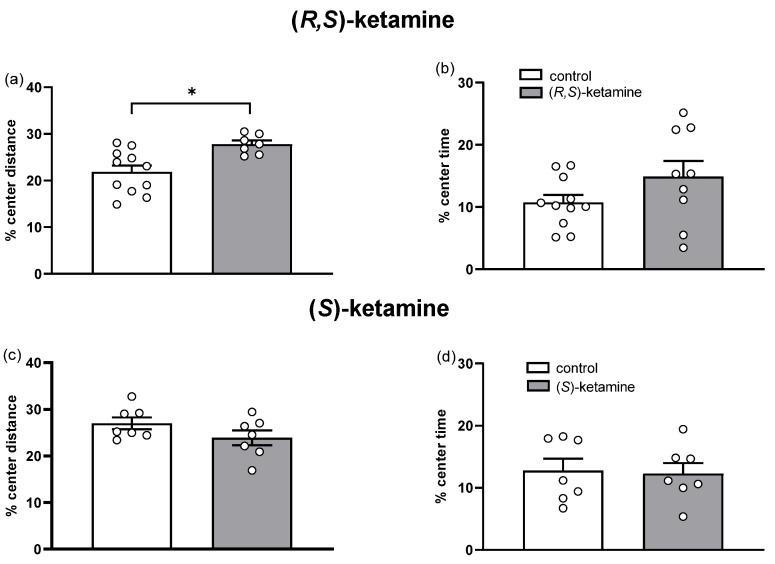
Effects of (*R*,*S*)-ketamine and (*S*)-ketamine (both 10 mg/kg, ip, 4 h before testing) on anxiety-related behaviors in stressed mice assessed in the open field test. Percentage of distance moved in and percentage of time spent in the center of the open field test in stressed mice treated with (*R*,*S*)-ketamine (**a**,**b**) and (*S*)-ketamine (**c**,**d**) in the first 10 min of observation. Bars represent mean ± SEM, while dots represent individual performance. Unpaired *t*-test: * *p* < 0.05 vs. control.

Anxiety-related behavior was assessed in the open field test through the percentage of time spent and the percentage of distance moved by the animal in the center of the apparatus in relation to the exploration of the whole arena (Figure 6). The treatment with (*R*,*S*)-ketamine increased the percentage of distance moved in the center (Figure 6a, *t*-test: t(16) = 3.2, *p* = 0.006) in the first 10 min of open field exploration in comparison with the control group, without significantly affecting the percentage of center time (Figure 6b, *p* > 0.05). By contrast, acute (*S*)-ketamine did not show any effect when compared to control, neither in the percentage of distance moved in the center nor in the percentage of time spent in the center (Figure 6c,d, *p* > 0.05). Still considering that increased spontaneous locomotion can influence the measurement of anxiety-related behaviors in the open field test, a correlation between the percentage of distance moved in the center and total distance moved in the open field test in (*R*,*S*)-ketamine-treated mice was calculated. However, the Pearson’s correlation did not show a significant relationship between total locomotion and exploration of the central area of the open field (r^2^ = 0.01, *p* = 0.99).

## 4. Discussion

In this study, we showed that in previously stressed mice, (*R*,*S*)- and (*S*)-ketamine acutely administered before re-exposition to the aversive stimulus did not affect memory retrieval of the avoidance behavior. Furthermore, (*S*)-ketamine reduced avoidance responses at the second part of the re-exposition session, while the racemic mixture tangentially did. In the tail suspension test, (*R*,*S*)- and (*S*)-ketamine similarly reduced immobility time, thus suggesting antidepressant-like effects. Additionally, only (*R*,*S*)-ketamine significantly increased the total distance moved in the open field and improved exploration to the center area. Together, these findings suggest that at the dose tested, acute administration of (*S*)-ketamine reduces avoidance responses during re-exposition to aversive stimuli and displays antidepressant effects. In some extension, the racemic mixture induced similar effects of (*S*)-ketamine in the active avoidance task, but with less efficacy. By contrast, only the racemic mixture reduced anxiety-related behaviors. Ultimately, these findings reinforce differences in the profile of action of (*R*,*S*)-ketamine and its (*S*)-enantiomer, being that (*R*,*S*)-ketamine is an agent with antidepressant and anxiolytic effects with weak ability to reduce avoidance responses.

The active avoidance task uses aversive stimuli (unconditioned, e.g., footshock) to create an associative memory. Usually, animals learn to escape from aversive stimuli by moving to the opposite side of a shuttle-box, then associating the arrival of the unpleasant stimuli with the light provided beforehand (e.g., conditioned stimuli) [25]. The understanding of the biological bases of avoidance memories and the ability of psychotropic drugs to modulate them are worth studying. Timing is a relevant aspect to be aware of when investigating the effects of drugs on aversive memories. For example, the effects of administering ketamine before acquisition of a task are clinically relevant for prophylactic treatments. By contrast, the effects of drug administration immediately after a training test mimics trauma analgesia, while a delayed dose of a drug after consolidation of a fear memory or prior to extinction training simulates a therapeutic modality to reopen the processing of reprogramming an avoidance information (for a review, see Choi et al. [1]).

This study tested the effects of acute doses of (*R*,*S*)- and (*S*)-ketamine administered 1 h before a re-exposition session of an active avoidance task in previously stressed mice. At this experimental condition, stressed mice retard the acquisition of an avoidance response. In this study, (*R*,*S*)-ketamine and its enantiomer did not affect the acquisition of avoidance responses when re-exposed to the presence of aversive stimuli. On the other hand, the racemic mixture, but especially its (*S*)-isomer, reduced avoidance responses in stressed mice. This scenario seems to be unrelated to the anxiolytic effects of (*R*,*S*)-ketamine, since the (*S*)-isoform significantly reduces avoidance responses without evoking anxiolysis.

In the present work, neither (*R*,*S*)-ketamine nor its (*S*)-enantiomer affected the retrieval of the conditioned avoidance response. In fact, in the re-exposition session, the control group increased the number of escapes and decreased the latencies of escapes from the electrified side of the box, while both forms of ketamine behaved similarly to controls, particularly in the first 15 footshocks. However, during the second part of the re-exposition session, (*R*,*S*)-ketamine-treated mice, but specially (*S*)-isomer, escaped less and more slowly compared to the behavior displayed in the first part of this session. On the contrary, control mice did not change their behavior in the second part of the test session compared to the first. It seems that the treatment with both forms of ketamine before the re-exposition to the aversive stimulus preserved the retrieval of the avoidance response, but at the same time, reduced the avoidance responses. Recent literature findings show that (*S*)-ketamine, at 5 mg/kg ip, immediately after, but not beyond 6 h of the re-exposure to the traumatic stimulus, significantly alleviates post-traumatic social avoidance in social defeat stressed mice [26]. In our study, we tested the effects of both forms of ketamine 1 h before re-exposition to a traumatic stimulus, and post-traumatic avoidance responses were ameliorated. Importantly, the present study investigated the effects of racemic mixture and (*S*)-ketamine on avoidance memories, with (*S*)-isoform being more effective in attenuating traumatic memories during re-exposition to aversive stimuli. Since we administered both forms of ketamine before re-exposure to the traumatic stimulus, our findings could be of interest in a ketamine-assisted psychotherapy session in PTSD patients.

This study used inescapable stress as stress inducer to compare the action between the enantiomer (*S*)- and the racemic ketamine in the tail suspension and open field tests. The protocol of stress used herein prompts the evocation of depressive- and anxiogenic-like behaviors [27,28]. (*R*,*S*)-ketamine and its (*S*)-enantiomer evoked equivalent antidepressant-like effects in the tail suspension test, as demonstrated by the lower immobility time compared to the controls when the animals were suspended by their tails. A number of studies have already pre-clinically tested the antidepressant effects of ketamine and its isoforms. However, this is the first to compare the effects of racemic mixture with (*S*)-ketamine in depression and concomitantly investigate the actions of these drugs in an active avoidance task. Ide et al. [29] observed antidepressant-like behaviors induced by racemic ketamine and the enantiomers (all injected at 10 mg/kg, ip) in mice in the tail suspension test. In a comparative study of the antidepressant effects of single intranasal injection of ketamine and its isoforms in mice, the order of potency of antidepressant effects was (*R*)-ketamine > (*R*,*S*)-ketamine > (*S*)-ketamine [30]. (*S*)-ketamine, in turn, also displayed antidepressant effects at acute doses in rodent animal models [15,16,17]. Together, these findings indicate that at the same dose used herein, racemic ketamine and its (*S*)-isoform are effective in reducing depressive-like behaviors in rats and mice, which is in line with the data reported here.

Anxiety-related behaviors were measured by the percentage of time and distance moved in the center of the open field, and only mice treated with racemic ketamine showed an increase in exploration to the center areas. Additionally, (*R*,*S*)-ketamine, but not (*S*)-, significantly increased spontaneous locomotion in mice. It is important to be aware that hyperlocomotion could potentially bias the interpretation of some behavioral tests, such as open field and tail suspension. However, in the present study, we did not find any significant correlation between the animal behavior in the tail suspension test and center arena exploration with the total distance moved in the open field. Therefore, our findings suggest that (*R*,*S*)-ketamine, but not (*S*)-ketamine, induces anxiolytic effects in mice by enhancing the exploration of unprotected environments.

Increased exploration to the center of the open field test represents a reduction in the conflict of staying in the periphery area (safer) versus exploring the central area of the apparatus (more exposed) [24]. The racemic mixture is most commonly reported in the literature as effective for anxiety, with the onset of effects occurring within the first 12 h after administration and long-term benefits extending up to 2 weeks [31]. Supporting the findings of our study, Fraga et al. [32] observed an anxiolytic effect of (*R*,*S*)-ketamine at 10 mg/kg in the open field, elevated plus maze, and light–dark preference test in female Swiss mice. Furthermore, according to a review study, of 17 studies using mice, 14 of them reported that acutely administered (*R*,*S*)-ketamine has anxiolytic effects, though few studies were found with (*S*)-ketamine [31]. In fact, of the three studies that tested the effects of (*S*)-ketamine on anxiety, only one [33] observed anxiolytic effects in rats [34,35]. In humans, some studies reported improvement in anxiety with acute (*S*)-ketamine treatment [36,37], while mostly stating that the racemic mixture is more effective in anxiety, as referenced in Hartland et al. [38]. Thus, similar to the literature, the present study also suggests a divergence between (*R*,*S*)- and (*S*)-ketamine as potential drugs to treat anxiety. 

Hyperlocomotion or motor incoordination can be among the main side effects of ketamine [39]. When evaluating the locomotor profile of our experimental groups, we observed that only (*R*,*S*)-ketamine evoked hyperlocomotion when assessed 4 h after drug administration. A relevant aspect to take into account is that locomotion was assessed in stressed mice. Another study has already reported that ketamine reverses the stress-induced reduction of locomotion [40]. Here, we did not evaluate the behavior of non-stressed controls compared to stressed mice. However, previous data from our research group showed that non-stressed controls move approximately 60,000 mm in the open field in 30 min [41]. In the present study, we observed that stressed controls moved around 40,000 mm in similar conditions to those in the above-mentioned study. Therefore, stress potentially reduced locomotion, and (*R*,*S*)-ketamine, but not the (*S*)-enantiomer, reversed this behavior. The ketamine-induced increased locomotion in the arena can be related to increased motivation and reduced anxiety-related behavior linked to an unfamiliar environment.

It is worth mentioning that in our study, both ketamine ((*R*,*S*)- and (*S*)-) were shown to equally improve depressive-like behaviors. However, the racemic mixture was divergent from (*S*)-ketamine in some aspects; in fact, it presented an anxiolytic profile along with a facilitated reduction of avoidance responses during re-exposition to an aversive stimulus. These differences may be due to the dose used: 10 mg/kg for both. Importantly, the racemic mixture ((*R*,*S*)-ketamine) contains equal parts of (*R*)-ketamine and (*S*)-ketamine (or esketamine), and the calculated Ki for the NMDA receptor in the crescent order is (*R*)-ketamine (Ki = 1.4 μM), (*R*,*S*)-ketamine (Ki = 0.53 μM), and (*S*)-ketamine (Ki = 0.30 μM) [42]. Therefore, at the molecular level, (*S*)-ketamine has four-fold greater binding affinity to NMDA receptors and twice the affinity for muscarinic receptors than (*R*)-ketamine [43]. Considering the biological actions of enantiomers, the racemic mixture contains the two isoforms of ketamine in a lower concentration compared to (*S*)-ketamine, and this may reflect in the divergence of biological actions observed in this study. In this context, the effects on avoidance responses may be more linked to the (*S*)-enantiomer, while the anxiolytic actions may be more related to the (*R*)-isoform. By contrast, both isoforms contribute to the antidepressant actions of ketamine.

## 5. Conclusions

Our findings show that acute (*S*)-ketamine, and to a lesser extent, the racemic mixture, at an antidepressant dose, did not affect avoidance memory retrieval, but reduced avoidance responses when injected 1 h before re-exposition to an aversive stimulus. Additionally, only (*R*,*S*)-ketamine exhibited an acute anxiolytic profile of action. Taken together, these behavioral findings reinforce the therapeutic potential of ketamine for the treatment of stress-related disorders, with (*R*,*S*)-ketamine at our experimental conditions exhibiting wider anti-stress properties than the S-enantiomer. Some limitations are present in this study, such as lack of prolonged behavioral observation of ketamine-treated mice in order to understand the time-course of ketamine’s effects on avoidance responses. The lack of electrophysiological and/or molecular data to reinforce the behavioral findings is also an important limitation to the present study. Taken together, this study provides novel results from a comparative scenario between (*S*)-ketamine and the racemic mixture and their effectiveness for treating stress-related disorders. Ultimately, the potential of ketamine to modify the expression of fear by reducing avoidance responses during re-exposition to aversive stimuli might enhance the efficacy of ketamine-assisted psychotherapy in individuals with chronic PTSD.

## Data Availability

The raw data supporting the conclusions of this article will be made available by the corresponding author on request.
